# Improving the Accuracy of Laplacian Estimation with Novel Variable Inter-Ring Distances Concentric Ring Electrodes

**DOI:** 10.3390/s16060858

**Published:** 2016-06-10

**Authors:** Oleksandr Makeyev, Walter G. Besio

**Affiliations:** 1 Department of Mathematics, Diné College, Tsaile, AZ 86556, USA; 2 Department of Electrical, Computer, and Biomedical Engineering, University of Rhode Island, Kingston, RI 02881, USA; besio@uri.edu

**Keywords:** noninvasive, electrophysiology, electroencephalography, sensors, multipolar, concentric ring electrodes, Laplacian, finite element method, modeling

## Abstract

Noninvasive concentric ring electrodes are a promising alternative to conventional disc electrodes. Currently, the superiority of tripolar concentric ring electrodes over disc electrodes, in particular, in accuracy of Laplacian estimation, has been demonstrated in a range of applications. In our recent work, we have shown that accuracy of Laplacian estimation can be improved with multipolar concentric ring electrodes using a general approach to estimation of the Laplacian for an (*n* + 1)-polar electrode with *n* rings using the (4*n* + 1)-point method for *n* ≥ 2. This paper takes the next step toward further improving the Laplacian estimate by proposing novel variable inter-ring distances concentric ring electrodes. Derived using a modified (4*n* + 1)-point method, linearly increasing and decreasing inter-ring distances tripolar (*n* = 2) and quadripolar (*n* = 3) electrode configurations are compared to their constant inter-ring distances counterparts. Finite element method modeling and analytic results are consistent and suggest that increasing inter-ring distances electrode configurations may decrease the truncation error resulting in more accurate Laplacian estimates compared to respective constant inter-ring distances configurations. For currently used tripolar electrode configuration, the truncation error may be decreased more than two-fold, while for the quadripolar configuration more than a six-fold decrease is expected.

## 1. Introduction

Electroencephalography (EEG) is an essential tool for brain and behavioral research, as well as one of the mainstays of hospital diagnostic procedures and pre-surgical planning. Despite scalp EEG’s many advantages, end users struggle with its poor spatial resolution, selectivity and low signal-to-noise ratio that are critically limiting the research discovery and diagnosis [1,2,3]. In particular, EEG’s poor spatial resolution is primarily due to (1) the blurring effects of the volume conductor with disc electrodes; and (2) EEG signals having reference electrode problems as idealized references are not available with EEG and interference on the reference electrode contaminates all other electrode signals [2]. The application of the surface Laplacian (the second spatial derivative of the potentials on the scalp surface) to EEG has been shown to alleviate the blurring effects enhancing the spatial resolution and selectivity, and reduce the reference problem [4,5,6].

**Figure 1:**
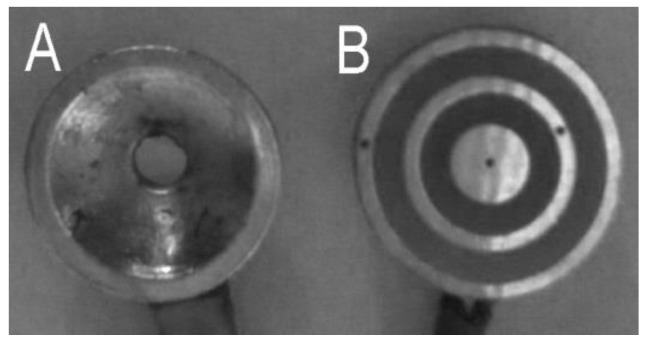
Conventional disc electrode (**A**) and tripolar concentric ring electrode (**B**).

Noninvasive concentric ring electrodes (CREs) can resolve the reference electrode problems since they act like closely spaced bipolar recordings [2]. They also act as spatial filters reducing the low spatial frequencies and increasing the spatial selectivity [7,8,9]. Moreover, CREs are symmetrical alleviating electrode orientation problems [9]. Most importantly, tripolar CREs (TCREs; Figure 1B) have been shown to estimate the surface Laplacian directly through the nine-point method, an extension of the five-point method (FPM) used for bipolar CREs, and significantly better than other electrode systems including bipolar and quasi-bipolar CRE configurations [10,11]. Compared to EEG with conventional disc electrodes (Figure 1A) Laplacian EEG via TCREs (tEEG) have been shown to have significantly better spatial selectivity (approximately 2.5 times higher), signal-to-noise ratio (approximately 3.7 times higher), and mutual information (approximately 12 times lower) [12]. Because of such unique capabilities, TCREs have found numerous applications in a wide range of areas including brain–computer interface [13,14], seizure onset detection [15,16], seizure attenuation using transcranial focal stimulation applied via TCREs [17,18,19,20], detection of high-frequency oscillations and seizure onset zones [21], *etc*. These EEG-related applications of TCREs, as well as recent applications related to electroenterograms [22,23], electrocardiograms [11,24,25,26], and electrohysterograms [27], suggest the potential of CRE technology in noninvasive electrophysiology, as well as the need for further improvement of CRE design.

Recent directions for such improvement include printing disposable TCREs on flexible substrates to increase the electrode's ability to adjust to body contours for better contact and to provide higher signal amplitude and signal-to-noise ratio [23,25,27], as well as assessing the effect of ring dimensions and electrode position on recorded signal [26]. However, the signal recorded from TCREs in References [23,25,26,27] is either a Laplacian derived for the case of the outer ring and the central disc of the TCRE being shorted together (quasi-bipolar CRE configuration) or just a set of bipolar signals representing differences between potentials recorded from the rings and the central disc. In our work, we are aiming to optimize the CRE design by combining the signals from all the recording surfaces available into a Laplacian estimate since for TCREs such approach has resulted in significantly higher Laplacian estimation accuracy and radial attenuation compared to bipolar and quasi-bipolar CRE configurations [10,11].

In Reference [28] we have shown that accuracy of Laplacian estimation can be improved with multipolar CREs. General approach to estimation of the Laplacian for an (*n* + 1)-polar electrode with *n* rings using the (4*n* + 1)-point method for *n* ≥ 2 has been proposed. This approach allows cancellation of all the Taylor series truncation terms up to the order of 2*n*, which has been shown to be the highest order achievable for a CRE with *n* rings [28]. Proposed approach was validated using finite element method (FEM) modeling. Multipolar concentric ring electrode configurations with *n* ranging from 1 ring (bipolar electrode configuration) to 6 rings (septapolar electrode configuration) were compared and obtained results suggested statistical significance of the increase in Laplacian accuracy caused by increase of the number of rings *n* [28].

To the best of the authors’ knowledge, all the previous research on CREs was based on the assumption of constant inter-ring distances (distances between consecutive rings). This means that distances between the rings were not considered as a means of improving the accuracy of Laplacian estimation. This paper takes the next fundamental step toward further improving the Laplacian estimation accuracy by proposing novel variable inter-ring distances CREs. Laplacian estimates for linearly increasing and linearly decreasing inter-ring distances TCRE (*n* = 2) and quadripolar CRE (QCRE; *n* = 3) configurations are derived using a modified (4*n* + 1)-point method from Reference [28] and directly compared to their constant inter-ring distances counterparts. Analytic analysis and FEM modeling are used to draw this comparison. Main results include establishing a connection between the analytic truncation term coefficient ratios from the Taylor series used in (4*n* + 1)-point method and respective ratios of Laplacian estimation errors obtained using the FEM model. Both ratios are consistent in suggesting that increasing inter-ring distances CRE configurations may offer more accurate Laplacian estimates compared to respective constant inter-ring distances CRE configurations. For currently used TCREs the Laplacian estimation error may be decreased more than two-fold, while, for the QCREs, more than six-fold decrease in estimation error is expected.

## 2. Materials and Methods

### 2.1. Notations and Preliminaries

**Figure 2:**
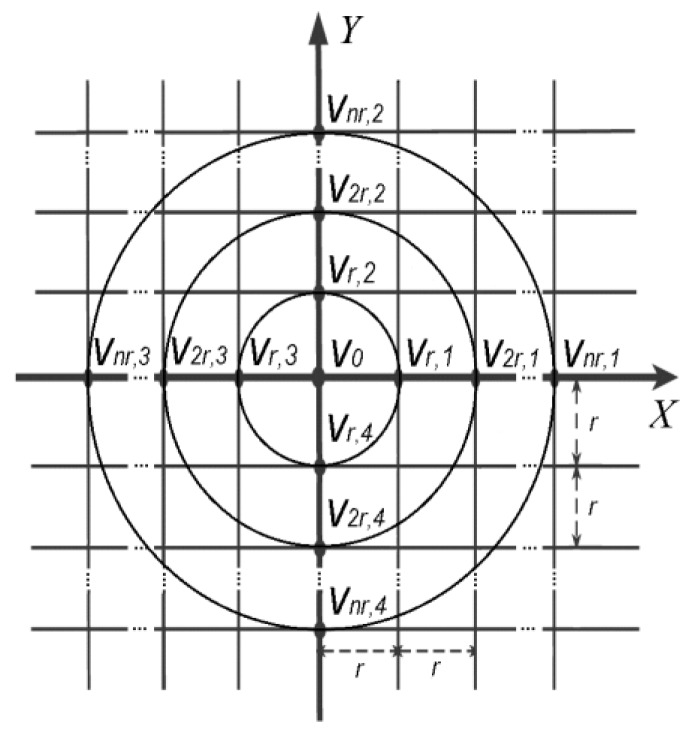
Regular plane square grid with inter-point distances equal to *r*.

In Reference [28] general (4*n* + 1)-point method for constant inter-ring distances (*n* + 1)-polar CRE with *n* rings was proposed. It was derived using a regular plane square grid with all inter-point distances equal to *r* presented in Figure 2.

First, FPM was applied to the points with potentials *v*_0_, *v_nr,_*_1_, *v_nr,_*_2_, *v_nr,_*_3_ and *v_nr,_*_4_ following Huiskamp’s calculation of the Laplacian potential ∆*v*_0_ using Taylor series [29]:

(1) $$\Delta v_{0}=\frac{\partial^{2}v}{\partial x^{2}}+\frac{\partial^{2}v}{\partial y^{2}}=\frac{1}{r^{2}}(\sum_{i=1}^{4}v_{r,i}-4v_{0})+O(r^{2})$$

where $O(r^{2})=\frac{r^{2}}{4!}(\frac{\partial^{4}v}{\partial x^{4}}+\frac{\partial^{4}v}{\partial y^{4}})+\frac{r^{4}}{6!}(\frac{\partial^{6}v}{\partial x^{6}}+\frac{\partial^{6}v}{\partial y^{6}})+...$ is the truncation error.

Equation (1) was generalized by taking the integral along the circle of radius *r* around point with potential *v*_0_. Defining *x = r*cos(*θ*) and *y = r*sin(*θ*) as in Huiskamp [29] we obtain:

(2) $$\frac{1}{2\pi }\int_{0}^{2\pi }v(r,\theta )d\theta -v_{0}=\frac{r^{2}}{4}\Delta v_{0}+\frac{r^{4}}{4!}\int_{0}^{2\pi }\sum_{j=0}^{4}\sin^{4-j}(\theta )\cos^{j}(\theta )d\theta (\frac{\partial^{4}v}{\partial x^{4-j}\partial y^{j}})+...$$

where $\frac{1}{2\pi }\int_{0}^{2\pi }v(r,\theta )d\theta$ is the average potential on the ring of radius *r* and *v*_0_ is the potential on the central disc of the CRE.

Next, a second FPM was applied with an integral along a circle of radius 2*r* (*v*_0_, *v*_2*r,*1_, *v*_2*r,*2_, *v*_2*r,*3_ and *v*_2*r,*4_ on Figure 2) around the point with potential *v*_0_ [10,11] producing the following for the difference between the average potential on the ring of radius 2*r* and the potential on the central disc of the CRE:

(3) $$\frac{1}{2\pi }\int_{0}^{2\pi }v(2r,\theta )d\theta -v_{0}=\frac{{\left(2r\right)}^{2}}{4}\Delta v_{0}+\frac{{\left(2r\right)}^{4}}{4!}\int_{0}^{2\pi }\sum_{j=0}^{4}\sin^{4-j}(\theta )\cos^{j}(\theta )d\theta (\frac{\partial^{4}v}{\partial x^{4-j}\partial y^{j}})+...$$

Finally, generalizing Equations (2) and (3) for a case of multipolar CRE with *n* rings (*n* ≥ 2) we obtain a set of *n* FPM equations, one for each ring with radii ranging from *r* to *nr* (*v*_0_, *v_nr,_*_1_, *v_nr,_*_2_, *v_nr,_*_3_ and *v_nr,_*_4_ on Figure 2) around the point with potential *v*_0_ [28]:

(4) $$\begin{array}{l} \frac{1}{2\pi }\int_{0}^{2\pi }v(nr,\theta )d\theta -v_{0}=\frac{{\left(nr\right)}^{2}}{4}\Delta v_{0}+\frac{{\left(nr\right)}^{4}}{4!}\int_{0}^{2\pi }\sum_{j=0}^{4}\sin^{4-j}(\theta )\cos^{j}(\theta )d\theta (\frac{\partial^{4}v}{\partial x^{4-j}\partial y^{j}})\\ +\frac{{\left(nr\right)}^{6}}{6!}\int_{0}^{2\pi }\sum_{j=0}^{6}\sin^{6-j}(\theta )\cos^{j}(\theta )d\theta (\frac{\partial^{6}v}{\partial x^{6-j}\partial y^{j}})+... \end{array}$$

To estimate the Laplacian for this general case the *n* equations are combined in a way that cancels all the truncation terms up to the highest order that can be achieved for *n* rings increasing the accuracy of the Laplacian estimate. In order to find such a combination we arrange the coefficients *l^k^* of the truncation terms with the general form $\frac{{\left(lr\right)}^{k}}{k!}\int_{0}^{2\pi }\sum_{j=0}^{k}\sin^{k-j}(\theta )\cos^{j}(\theta )d\theta (\frac{\partial^{k}v}{\partial x^{k-j}\partial y^{j}})$ for order *k* ranging in increments of 2 from 4 to 2*n* and ring radius multiplier *l* ranging from 1 (Equation (2)) to *n* (Equation (4)) into an *n* − 1 by *n* matrix *A* that is a function only of the number of the rings *n*:

(5) $$A=\left(\begin{array}{cccc} 1 & 2^{4} & \cdots & n^{4}\\ 1 & 2^{6} & \cdots & n^{6}\\ \vdots & \vdots & \ddots & \vdots \\ 1 & 2^{2n} & \cdots & n^{2n} \end{array}\right)$$

A matrix equation of the form:

(6) $$A\bar{x}=\bar{0}$$

is equivalent to a homogeneous system of linear equations where $\bar{0}$ is the (*n* − 1)-dimensional zero vector and $\bar{x}$ is the *n*-dimensional vector that allows the cancellation of all the truncation terms up to the order of 2*n* by setting the linear combination of *n* coefficients *l^k^* corresponding to all ring radii for each order *k* equal to 0 [28]. We have showed that 2*n* is the highest truncation term order that can be cancelled out for a CRE with *n* rings while assuring existence of nontrivial solution ($\bar{x}\neq \bar{0}$) of Equation (6) by keeping the homogeneous system underdetermined [28].

Solution $\bar{x}$ of Equation (6) is given by the null space (or kernel) of matrix *A* [30]. Moreover, it should be noted that such null space vectors used for Laplacian estimates are not unique. From the properties of matrix multiplication it follows that for any vector $\bar{x}$ that belongs to the null space of matrix *A* and a scalar *c* the scaled vector $c\bar{x}$ also belongs to the null space of the same matrix *A* since $\left(cA)\bar{x}=c(A\bar{x}\right)$. Therefore, any scaled version of given null space vector would also be a null space vector.

### 2.2. Variable (Linearly Increasing and Linearly Decreasing) Inter-Ring Distances CREs

We consider the case of CRE configurations with variable inter-ring distances that increase or decrease linearly the further the concentric ring lies from the central disc. To modify the (4*n* + 1)-point method from Reference [28] to the case of linearly increasing inter-ring distances, the distance between the central point with potential *v*_0_ and four points on the first concentric ring (the smallest and the closest one to the central point) is set equal to *r*. The distance between the first and the second (second closest to the central point) concentric ring is set equal to 2*r*. The distance between the second and the third (third closest to the central point) concentric ring is set equal to 3*r*, *etc*. In this case, the sum of all the inter-ring distances to the outer (furthest from the central point), *n*-th, ring can be obtained using the formula for the *n*-th term of the triangular number sequence that describes the sum of all points in a triangular grid where the first row contains a single point and each subsequent row contains one more point than the previous one to be equal to *n*(*n* + 1)/2 [31]. Therefore, modified matrix *A* of truncation term coefficients *l^k^* from Equation (5) for linearly increasing inter-ring distances CRE is equal to:

(7) $$A^{\prime }=\left(\begin{array}{cccc} 1 & 3^{4} & \cdots & {\left(\frac{n(n+1)}{2}\right)}^{4}\\ 1 & 3^{6} & \cdots & {\left(\frac{n(n+1)}{2}\right)}^{6}\\ \vdots & \vdots & \ddots & \vdots \\ 1 & 3^{2n} & \cdots & {\left(\frac{n(n+1)}{2}\right)}^{2n} \end{array}\right)$$

In the opposite case of CRE configuration with inter-ring distances decreasing linearly the further the concentric ring lies from the central disc the distance between the outer (furthest from the central point), *n*-th, concentric ring and the second to last (second furthest from the central point) concentric ring is equal to *r*. The distance between the second to last and the third to last (third furthest from the central point) concentric rings is set equal to 2*r*, *etc.* In this case, the sum of all the inter-ring distances preceding the outer, *n*-th, ring can also be found using the formula for the *n*-th term of the triangular number sequence due to the commutative property of addition. Therefore, modified matrix *A* of truncation term coefficients *l^k^* from Equation (5) for linearly decreasing inter-ring distances CRE is equal to:

(8) $$A^{\prime \prime }=\left(\begin{array}{cccc} n^{4} & {\left(2n-1\right)}^{4} & \cdots & {\left(\frac{n(n+1)}{2}\right)}^{4}\\ n^{6} & {\left(2n-1\right)}^{6} & \cdots & {\left(\frac{n(n+1)}{2}\right)}^{6}\\ \vdots & \vdots & \ddots & \vdots \\ n^{2n} & {\left(2n-1\right)}^{2n} & \cdots & {\left(\frac{n(n+1)}{2}\right)}^{2n} \end{array}\right)$$

**Figure 3:**
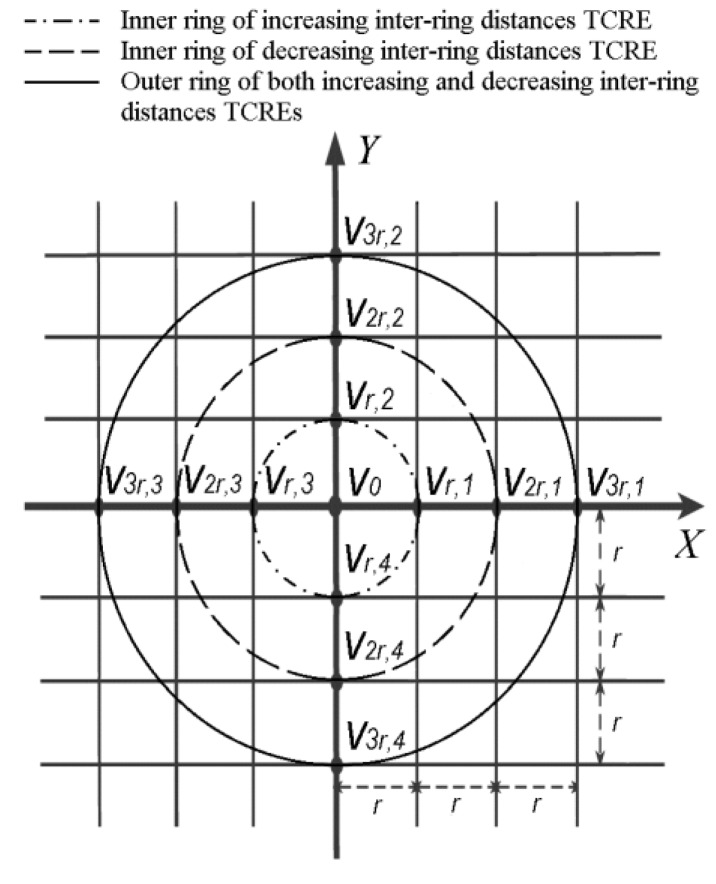
Linearly increasing and linearly decreasing inter-ring distances TCREs.

An example including both linearly increasing and linearly decreasing inter-ring distances TCREs is presented in Figure 3.

### 2.3. FEM Modeling

**Figure 4:**
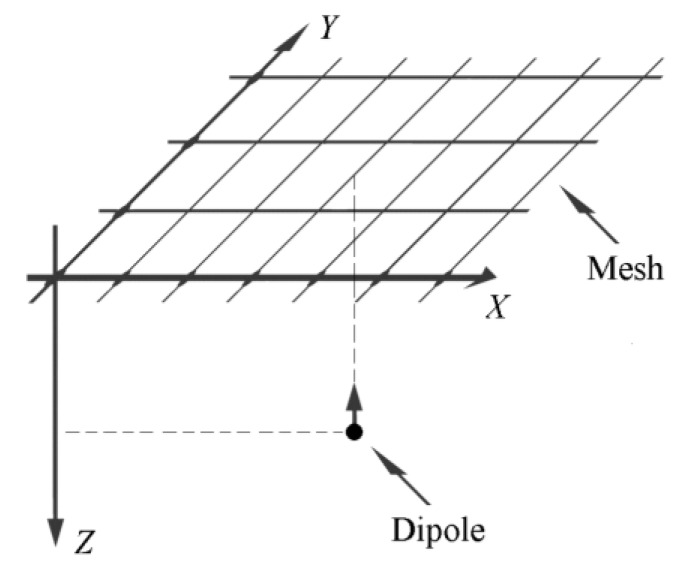
Schematic of the FEM model with an evenly spaced square mesh size of 600 × 600 used to assess and compare the accuracy of Laplacian estimates for constant and variable inter-ring distances CRE configurations.

All the FEM modeling was performed using Matlab (Mathworks, Natick, MA, USA). To directly compare the discrete Laplacian estimates including the previously proposed constant inter-ring distances TCRE (*n* = 2) and QCRE (*n* = 3) configurations to their counterparts with variable inter-ring distances a FEM model from References [10,11,28] was used with an evenly spaced square mesh size of 600 × 600 located in the first quadrant of the *X*-*Y* plane above a unit charge dipole projected to the center of the mesh and oriented towards the positive direction of the *Z* axis as shown in Figure 4.

**Figure 5:**
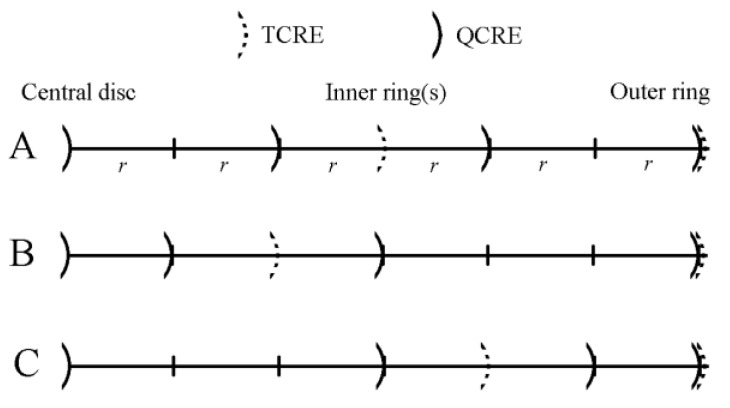
Relative locations of rings, with respect to the central disc, for constant inter-ring distances (**A**), increasing inter-ring distances (**B**), and decreasing inter-ring distances (**C**) TCRE and QCRE configurations.

Namely, comparisons to the linearly increasing and linearly decreasing variable inter-ring distances TCRE and QCRE configurations respectively were drawn. Bipolar CRE configuration (*n* = 1) was also included in the FEM model. To ensure direct comparability of results for different CRE configurations, all modeled bipolar, tripolar and quadripolar CREs had the same dimensions despite having different numbers of rings. The largest, outer ring radius for all the CRE configurations was selected to be equal to 6*r* since 6 is the least common multiple of 2 and 3. Relative locations of concentric rings for all the TCRE and QCRE configurations modeled are presented in Figure 5. The outer ring for the bipolar CRE (the only concentric ring in this configuration) coincides with the outer rings for TCRE and QCRE configurations (Figure 5).

At each point of the mesh, the electric potential *ϕ* generated by a unity dipole was calculated with the formula for electric potential due to a dipole in a homogeneous medium of conductivity *σ* [32]:

(9) $$\phi =\frac{1}{4\pi \sigma }\frac{({\bar{r}}_{p}-\bar{r})\cdot \bar{p}}{{\left|{\bar{r}}_{p}-\bar{r}\right|}^{3}}$$

where $\bar{r}=(x,y,z)$ and $\bar{p}=(p_{x},p_{y},p_{z})$ represent the location and the moment of the dipole and ${\bar{r}}_{p}=(x_{p},y_{p},z_{p})$ represents the observation point. The conductivity *σ* of the medium was taken to be 7.14 ms/cm to emulate biological tissue [33]. For this FEM model it was assumed that the medium was homogeneous and $\bar{p}=(0,\;0,\;1)$ making the term $\bar{p}/4\pi \sigma$ in Equation (9) constant. The analytical Laplacian was then calculated at each point of the mesh, by taking the second derivative of the electric potential *ϕ* [32]:

(10) $$L=\Delta \phi =\frac{\partial^{2}\phi }{\partial x^{2}}+\frac{\partial^{2}\phi }{\partial y^{2}}$$

According to He and Wu [32], this results in:

(11) $$L=\frac{3}{4\pi \sigma }\left[5{\left(z_{p}-z\right)}^{2}\frac{({\bar{r}}_{p}-\bar{r})\cdot \bar{p}}{{\left|{\bar{r}}_{p}-\bar{r}\right|}^{7}}-\frac{({\bar{r}}_{p}-\bar{r})\cdot \bar{p}+2(z_{p}-z)p_{z}}{{\left|{\bar{r}}_{p}-\bar{r}\right|}^{5}}\right]$$

Laplacian estimates for seven CRE configurations were computed at each point of the mesh where appropriate boundary conditions could be applied. Modeling was repeated for different integer multiples of *r* ranging from 1 to 10. Therefore, in the worst case scenario of a CRE being modeled with the inter-point distance using a multiple value equal to 10 the number of points on the mesh where appropriate boundary conditions could be applied to compute Laplacian estimates was equal to 480 × 480 (since for each dimension of the mesh 600 − 2 × 6 × 10 = 480). Correspondingly, in the best case scenario for a multiple value equal to 1 the number of points on the mesh where Laplacian estimates were computed was equal to 588 × 588 (600 − 2 × 6 × 1 = 588). Since the model was tied to the physical dimensions (in cm) through the target physical size of the CRE, the smallest CRE diameter was equal to 0.5 cm (multiple of *r* equal to 1) and the largest was equal to 5 cm (multiple of *r* equal to 10). The dipole depth was equal to 5 cm.

Derivation of Laplacian estimate coefficients for variable inter-ring distances CRE configurations was performed using the approach proposed in this paper by finding the null space of respective matrices *A*′ (Equation (7)) and *A*″ (Equation (8)) for *n* = 2 and *n* = 3. For TCREs the coefficients were (81, −1) and (81, −16) for increasing and decreasing inter-ring distances respectively. For QCREs the coefficients were (4374, −70, 1) and (6875, −2187, 625) for increasing and decreasing inter-ring distances, respectively. Coefficients for constant inter-ring distances CRE configurations were adopted from Reference [28]: (16, −1) for TCRE and (270, −27, 2) for QCRE. These seven estimates including three for TCRE (with constant, increasing, and decreasing inter-ring distances respectively), three for QCRE, and one for bipolar CRE configuration were then compared with the calculated analytical Laplacian for each point of the mesh where corresponding Laplacian estimates were computed using Relative Error and Maximum Error measures [10,11,28,29]:

(12) $${\text{Relative Error}}^{i}=\sqrt{\frac{\sum (\Delta v-\Delta^{i}v{)}^{2}}{\sum {\left(\Delta v\right)}^{2}}}$$

(13) $${\text{Maximum Error}}^{i}=\max\left|\Delta v-\Delta^{i}v\right|$$

where *i* represents the seven Laplacian estimation methods used to approximate the Laplacian potential Δ*^i^v* and Δ*v* represents the analytical Laplacian potential.

## 3. Results

### 3.1. FEM Modeling

**Figure 6:**
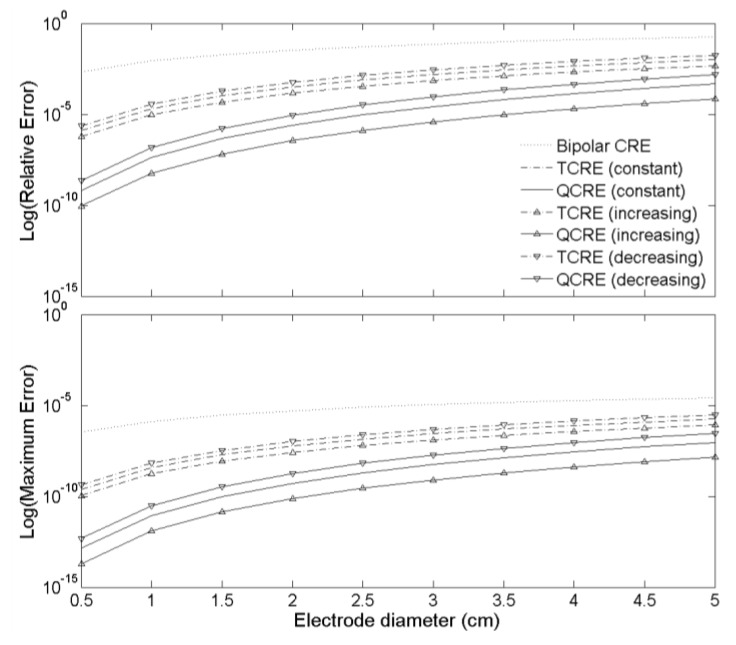
Relative (**Top**) and Maximum (**Bottom**) Errors of seven Laplacian estimates corresponding to bipolar, TCRE, and QCRE configurations.

The FEM modeling results of two error measures computed for seven Laplacian estimation methods corresponding to seven CRE configurations using Equations (12) and (13), respectively, are presented on a semi-log scale in Figure 6 for CRE diameters ranging from 0.5 cm to 5 cm.

Laplacian estimation errors in Figure 6 suggest that the increasing inter-ring distances TCRE and QCRE configurations hold potential for an improvement over their constant inter-ring distances counterparts while the decreasing inter-ring distances TCRE and QCRE configurations do not. Moreover, this improvement appears to become more significant with the increase of the number of rings (*i.e.*, there is more significant improvement for QCREs than for TCREs). This stems from comparison of averages (mean ± standard deviation for 10 different sizes of each CRE configuration) of errors for constant inter-ring distances and increasing inter-ring distances CREs. For TCREs Relative and Maximum Errors are 2.23 ± 0.02 and 2.22 ± 0.03 times higher on average for constant inter-ring distances CREs, respectively, while for QCREs Relative and Maximum Errors are 6.95 ± 0.14 and 6.91 ± 0.16 times higher on average for constant inter-ring distances CREs, respectively. Similar comparison of averages can be drawn for decreasing inter-ring distances and constant inter-ring distances CREs. For TCREs Relative and Maximum Errors are 1.75 ± 0.02 and 1.74 ± 0.03 times higher on average for decreasing inter-ring distances CREs, respectively, while for QCREs Relative and Maximum Errors are 3.41 ± 0.09 and 3.38 ± 0.11 times higher on average for decreasing inter-ring distances CREs, respectively.

It should be noted that these averages are presented for the FEM model with dipole depth of 5 cm. This dipole depth was selected since out of the range of dipole depths (1 cm to 5 cm) that were assessed in Reference [28], it corresponded to the smallest standard deviation values. The smallest standard deviation assures that Relative and Maximum Errors for 10 different sizes of each CRE configuration are as close as possible to the reported means.

### 3.2. Analytic Verification

Variable inter-ring distances CREs have the same number of rings and, therefore, the same number and order of truncation terms in Laplacian estimates as their constant inter-ring distances counterparts. Therefore, constant and variable inter-ring distances CRE configurations can be directly compared by assessing the coefficients at the respective truncation terms that comprise the truncation error of the Laplacian estimation.

Analyzing those coefficients will allow us to determine which electrode configuration allows minimizing the truncation error resulting in more accurate Laplacian estimate. Performing this kind of analysis for increasing and constant inter-ring distances as well as for constant and decreasing inter-ring distances TCREs and QCREs would allow verifying the results obtained by FEM modeling analytically.

#### 3.2.1. Increasing and Constant Inter-Ring Distances TCREs and QCREs

First, we derive the coefficients of the truncation terms for TCRE and QCRE configurations with increasing and constant inter-ring distances as functions of the order of the truncation term, *k*, under the conditions of the FEM model used in this study: the largest, outer ring radius equals to 6*r* and relative locations of concentric rings are as shown in Figure 5.

For constant inter-ring distances TCREs the coefficients used to combine the differences between the concentric ring potentials and the central disc potential into a Laplacian estimate can be derived using the approach proposed in Reference [28]. This approach cancels all the truncation terms up to the order of 2*n* which has been shown to be the highest order achievable for a CRE with *n* rings [28]. In the case of TCREs (*n* = 2) this corresponds to cancellation of the fourth order leaving truncation terms of orders 6 and higher. Assuming that our TCRE has two rings with radii *αr* and *βr*, respectively, such that *β* > *α*, for each ring we take the integral along the circle with the corresponding radius of the Taylor series in a manner identical to deriving Equations (2)–(4) to obtain:

(14) $$\begin{array}{l} \frac{1}{2\pi }\int_{0}^{2\pi }v\left(\alpha r,\theta \right)d\theta =v_{0}+\frac{{\left(\alpha r\right)}^{2}}{4}\Delta v_{0}+\frac{{\left(\alpha r\right)}^{4}}{4!}\int_{0}^{2\pi }\sum_{j=0}^{4}\sin^{4-j}\left(\theta \right)\cos^{j}\left(\theta \right)d\theta \left(\frac{\partial^{4}v}{\partial x^{4-j}\partial y^{j}}\right)\\ +\frac{{\left(\alpha r\right)}^{6}}{6!}\int_{0}^{2\pi }\sum_{j=0}^{6}\sin^{6-j}\left(\theta \right)\cos^{j}\left(\theta \right)d\theta \left(\frac{\partial^{6}v}{\partial x^{6-j}\partial y^{j}}\right)+... \end{array}$$

and:

(15) $$\begin{array}{l} \frac{1}{2\pi }\int_{0}^{2\pi }v\left(\beta r,\theta \right)d\theta =v_{0}+\frac{{\left(\beta r\right)}^{2}}{4}\Delta v_{0}+\frac{{\left(\beta r\right)}^{4}}{4!}\int_{0}^{2\pi }\sum_{j=0}^{4}\sin^{4-j}\left(\theta \right)\cos^{j}\left(\theta \right)d\theta \left(\frac{\partial^{4}v}{\partial x^{4-j}\partial y^{j}}\right)\\ +\frac{{\left(\beta r\right)}^{6}}{6!}\int_{0}^{2\pi }\sum_{j=0}^{6}\sin^{6-j}\left(\theta \right)\cos^{j}\left(\theta \right)d\theta \left(\frac{\partial^{6}v}{\partial x^{6-j}\partial y^{j}}\right)+... \end{array}$$

For constant inter-ring distances TCREs we combine Equations (14) and (15) by multiplying Equation (14) by 16, multiplying Equation (15) by −1, and adding the two resulting products together solving the sum for the Laplacian ∆*v*_0_:

(16) $$\begin{array}{l} \Delta v_{0}=\frac{1}{\left(\frac{16\alpha^{2}-\beta^{2}}{4}\right)r^{2}}[16\left(v_{MR}-v_{0}\right)-\left(v_{OR}-v_{0}\right)\\ +\frac{\left(16\alpha^{4}-\beta^{4}\right)r^{4}}{4!}\int_{0}^{2\pi }\sum_{j=0}^{4}\sin^{4-j}\left(\theta \right)\cos^{j}\left(\theta \right)d\theta \left(\frac{\partial^{4}v}{\partial x^{4-j}\partial y^{j}}\right)\\ +\frac{\left(16\alpha^{6}-\beta^{6}\right)r^{6}}{6!}\int_{0}^{2\pi }\sum_{j=0}^{6}\sin^{6-j}\left(\theta \right)\cos^{j}\left(\theta \right)d\theta \left(\frac{\partial^{6}v}{\partial x^{6-j}\partial y^{j}}\right)+...] \end{array}$$

where $v_{MR}=\frac{1}{2\pi }\int_{0}^{2\pi }v\left(\alpha r,\theta \right)d\theta$ is the potential on the middle ring of the radius *αr* and $v_{OR}=\frac{1}{2\pi }\int_{0}^{2\pi }v\left(\beta r,\theta \right)d\theta$ is the potential on the outer ring of the radius *βr*.

For increasing inter-ring distances TCREs, Equations (14) and (15) have to be combined with the coefficients 81 and −1, respectively, resulting in:

(17) $$\begin{array}{l} \Delta v_{0}=\frac{1}{\left(\frac{81\alpha^{2}-\beta^{2}}{4}\right)r^{2}}[81\left(v_{MR}-v_{0}\right)-\left(v_{OR}-v_{0}\right)\\ +\frac{\left(81\alpha^{4}-\beta^{4}\right)r^{4}}{4!}\int_{0}^{2\pi }\sum_{j=0}^{4}\sin^{4-j}\left(\theta \right)\cos^{j}\left(\theta \right)d\theta \left(\frac{\partial^{4}v}{\partial x^{4-j}\partial y^{j}}\right)\\ +\frac{\left(81\alpha^{6}-\beta^{6}\right)r^{6}}{6!}\int_{0}^{2\pi }\sum_{j=0}^{6}\sin^{6-j}\left(\theta \right)\cos^{j}\left(\theta \right)d\theta \left(\frac{\partial^{6}v}{\partial x^{6-j}\partial y^{j}}\right)+...] \end{array}$$

Both Laplacian Equations (16) and (17) allow cancellation of the fourth order truncation term since (16*α*^4^ − *β*^4^) is equal to 0 for *α* and *β* equal to 3 and 6, respectively (constant inter-ring distances TCRE; panel A of Figure 5), and (81*α*^4^ − *β*^4^) is equal to 0 for *α* and *β* equal to 2 and 6, respectively (increasing inter-ring distances TCRE; panel B of Figure 5).

Now we can express the coefficients *c*(*k*) of truncation terms with the general form $\frac{c\left(k\right)r^{k-2}}{k!}\int_{0}^{2\pi }\sum_{j=0}^{k}\sin^{k-j}\left(\theta \right)\cos^{j}\left(\theta \right)d\theta \left(\frac{\partial^{k}v}{\partial x^{k-j}\partial y^{j}}\right)$ as the function of the truncation term order *k*. For constant inter-ring distances TCRE $c_{C}^{TCRE}(k)=\frac{4\left(16\alpha^{k}-\beta^{k}\right)}{16\alpha^{2}-\beta^{2}}$ or, for *α* and *β* equal to 3 and 6, respectively, as defined in the FEM model, $c_{C}^{TCRE}(k)=\frac{4\left(16\cdot 3^{k}-6^{k}\right)}{16\cdot 3^{2}-6^{2}}=\;\frac{16\cdot 3^{k}-6^{k}}{27}$ for even *k* ≥ 6. For increasing inter-ring distances TCRE $c_{I}^{TCRE}(k)=\frac{4\left(81\alpha^{k}-\beta^{k}\right)}{81\alpha^{2}-\beta^{2}}$ or, for *α* and *β* equal to 2 and 6, respectively, as defined in the FEM model, $c_{I}^{TCRE}(k)=\frac{4\left(81\cdot 2^{k}-6^{k}\right)}{81\cdot 2^{2}-6^{2}}=\;\frac{81\cdot 2^{k}-6^{k}}{72}$ for even *k* ≥ 6.

The same steps can be taken to derive the truncation term coefficient functions for increasing and constant inter-ring distances QCREs (*n* = 3) cancelling the truncation terms up to the sixth order. For constant inter-ring distances QCRE coefficients (270, −27, 2) are used to combine potentials on three rings with radii 2*r*, 4*r*, and 6*r* (constant inter-ring distances QCRE; panel A of Figure 5) and the central disc resulting in $c_{C}^{QCRE}(k)=\frac{4\left(270\cdot 2^{k}-27\cdot 4^{k}+2\cdot 6^{k}\right)}{270\cdot 2^{2}-27\cdot 4^{2}+2\cdot 6^{2}}=\;\frac{270\cdot 2^{k}-27\cdot 4^{k}+2\cdot 6^{k}}{180}$ for even *k* ≥ 8. For increasing inter-ring distances QCRE coefficients (4374, −70, 1) are used to combine potentials on three rings with radii *r*, 3*r*, and 6*r* (increasing inter-ring distances QCRE; panel B of Figure 5) and the central disc resulting in $c_{I}^{QCRE}(k)=\frac{4\left(4374\cdot 1^{k}-70\cdot 3^{k}+1\cdot 6^{k}\right)}{4374\cdot 1^{2}-70\cdot 3^{2}+1\cdot 6^{2}}=\;\frac{4374-70\cdot 3^{k}+6^{k}}{945}$ for even *k* ≥ 8.

We hypothesize that the ratios of constant inter-ring distances truncation term coefficient functions over the increasing inter-ring distances truncation term coefficient functions calculated for respective TCRE and QCRE configurations will be comparable to the respective ratios of Relative and Maximum Errors obtained using the FEM model.

The ratio of truncation term coefficient functions for constant inter-ring distances to increasing inter-ring distances TCRE configurations is the following for even *k* ≥ 6:

(18) $$r_{CI}^{TCRE}(k)=\;\frac{c_{C}^{TCRE}(k)}{c_{I}^{TCRE}(k)}=\left(\frac{16\cdot 3^{k}-6^{k}}{27}\right)/\left(\frac{81\cdot 2^{k}-6^{k}}{72}\right)=\frac{8\left(16\cdot 3^{k}-6^{k}\right)}{3\left(81\cdot 2^{k}-6^{k}\right)}$$

In a similar way, the ratio of truncation term coefficient functions for constant inter-ring distances to increasing inter-ring distances QCRE configurations is the following for even *k* ≥ 8:

(19) $$r_{CI}^{QCRE}(k)=\frac{c_{C}^{QCRE}(k)}{c_{I}^{QCRE}(k)}=\frac{21\left(270\cdot 2^{k}-27\cdot 4^{k}+2\cdot 6^{k}\right)}{4\left(4374-70\cdot 3^{k}+6^{k}\right)}$$

**Figure 7:**
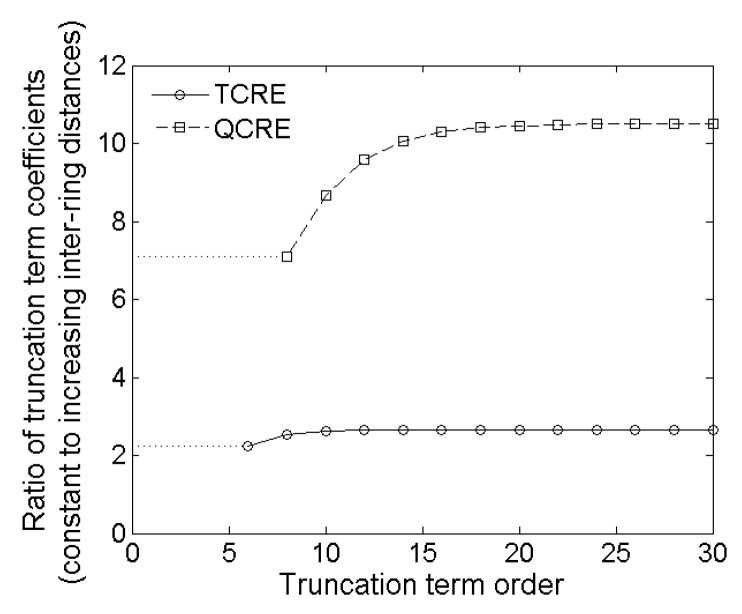
Ratios of constant inter-ring distances to increasing inter-ring distances truncation term coefficients as functions of the truncation term order for TCRE and QCRE configurations.

Plots of both functions from Equations (18) and (19) are presented in Figure 7 for even truncation term order *k* ranging from 6 to 30 and from 8 to 30, respectively.

While the signs of the truncation term coefficients are consistent for both constant and increasing inter-ring distances CRE configurations (all negative for TCREs and all positive for QCREs), Figure 7 serves a three-fold purpose. First, it shows that absolute values of coefficients are larger for constant inter-ring distances CRE configurations since ratios of truncation term coefficients for constant inter-ring distances CRE configurations over corresponding increasing inter-ring distances CRE configurations are all larger than 1. Second, Figure 7 shows that the ratios of truncation term coefficients are higher for QCREs than for TCREs. Therefore, the improvement in Laplacian accuracy is likely to become more significant with the increase in the number of rings. Third, Figure 7 shows that all the coefficient ratios increase with the increase of the truncation term order but according to Reference [34] for Taylor series “higher-order terms usually contribute negligibly to the final sum and can be justifiably discarded”. Therefore, we will consider the coefficient ratios for the lowest nonzero truncation term for TCRE (sixth order) and QCRE (eighth order) configurations equal to 2.25 and 7.11, respectively (dotted lines in Figure 7), as the ones that contribute the most to the truncation error. These analytically obtained ratios are comparable (difference of less than 5%) to the respective ratios of Relative and Maximum Errors obtained using the FEM model (Figure 6) for tripolar (2.23 ± 0.02 and 2.22 ± 0.03, respectively) and quadripolar (6.95 ± 0.14 and 6.91 ± 0.16, respectively) CRE configurations. Even if we take weighted arithmetic means of all the truncation term coefficient ratios from Figure 7 for truncation term orders up to 30 with weights derived from an exponential decay model with unit original amount and decay rate equal to −1 to account for decreasing contribution of higher order terms we obtain weighted average ratios of 2.37 and 7.83, respectively. These analytic ratios are still within 20% of the respective ratios of Relative and Maximum Errors obtained by FEM modeling.

#### 3.2.2. Constant and Decreasing Inter-Ring Distances TCREs and QCREs

In a manner identical to the one used in increasing inter-ring distances CREs we can show that for decreasing inter-ring distances TCRE $c_{D}^{TCRE}(k)=\frac{4\left(81\alpha^{k}-16\beta^{k}\right)}{81\alpha^{2}-16\beta^{2}}$ or, for *α* and *β* equal to 4 and 6, respectively (decreasing inter-ring distances TCRE; panel C of Figure 5), as defined in the FEM model, $c_{D}^{TCRE}(k)=\frac{4\left(81\cdot 4^{k}-16\cdot 6^{k}\right)}{81\cdot 4^{2}-16\cdot 6^{2}}=\;\frac{81\cdot 4^{k}-16\cdot 6^{k}}{180}$ for even *k* ≥ 6. For decreasing inter-ring distances QCRE coefficients (6875, −2187, 625) are used to combine potentials on three rings with radii 3*r*, 5*r*, and 6*r* (decreasing inter-ring distances QCRE; panel C of Figure 5) and the central disc resulting in $c_{D}^{QCRE}(k)=\frac{4\left(6875\cdot 3^{k}-2187\cdot 5^{k}+625\cdot 6^{k}\right)}{6875\cdot 3^{2}-2187\cdot 5^{2}+625\cdot 6^{2}}=\;\frac{6875\cdot 3^{k}-2187\cdot 5^{k}+625\cdot 6^{k}}{7425}$ for even *k* ≥ 8.

The ratio of truncation term coefficient functions for decreasing inter-ring distances to constant inter-ring distances TCRE configurations is the following for even *k* ≥ 6:

(20) $$r_{DC}^{TCRE}(k)=\;\frac{c_{D}^{TCRE}(k)}{c_{C}^{TCRE}(k)}=\;\left(\frac{81\cdot 4^{k}-16\cdot 6^{k}}{180}\right)/\left(\frac{16\cdot 3^{k}-6^{k}}{27}\right)=\frac{3\left(81\cdot 4^{k}-16\cdot 6^{k}\right)}{20\left(16\cdot 3^{k}-6^{k}\right)}$$

In a similar way, the ratio of truncation term coefficient functions for decreasing inter-ring distances to constant inter-ring distances QCRE configurations is the following for even *k* ≥ 8:

(21) $$r_{DC}^{QCRE}(k)=\;\;\frac{c_{D}^{QCRE}(k)}{c_{C}^{QCRE}(k)}=\frac{4\left(6875\cdot 3^{k}-2187\cdot 5^{k}+625\cdot 6^{k}\right)}{165\left(270\cdot 2^{k}-27\cdot 4^{k}+2\cdot 6^{k}\right)}$$

**Figure 8:**
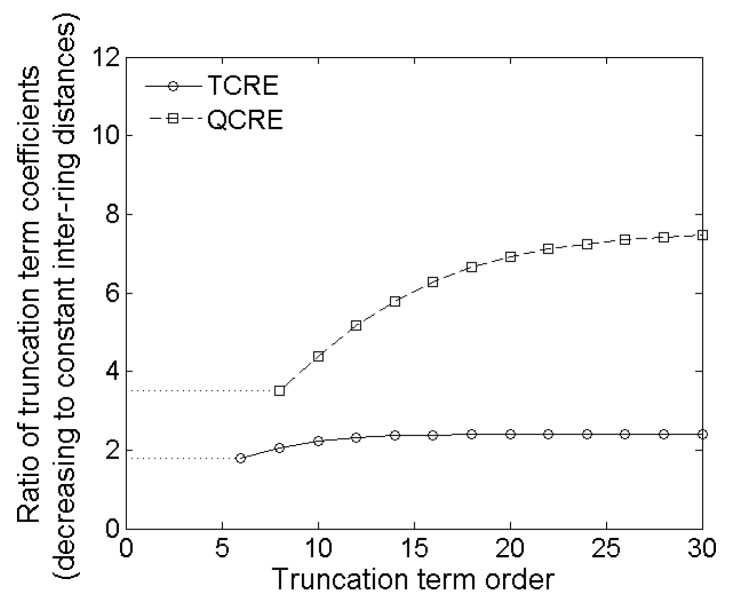
Ratios of decreasing inter-ring distances to constant inter-ring distances truncation term coefficients as functions of the truncation term order for TCRE and QCRE configurations.

Plots of both functions from Equations (20) and (21) are presented in Figure 8 for even truncation term order *k* ranging from 6 to 30 and from 8 to 30, respectively.

Similar conclusions to the ones derived from Figure 7 can be derived from Figure 8. Figure 8 suggests that truncation errors for decreasing inter-ring distances CRE configurations are greater than the ones for corresponding constant inter-ring distances CRE configurations which results in more accurate Laplacian estimates for constant inter-ring distances CRE configurations with the extent of improvement related to increase in the number of rings. More importantly, coefficient ratios for the lowest nonzero truncation term for TCRE (sixth order) and QCRE (eighth order) configurations are equal to 1.78 and 3.52, respectively (dotted lines in Figure 8). These analytically obtained ratios are again comparable (difference of less than 5%) to the respective ratios of Relative and Maximum Errors obtained using the FEM model for tripolar (1.75 ± 0.02 and 1.74 ± 0.03, respectively) and quadripolar (3.41 ± 0.09 and 3.38 ± 0.11, respectively) CRE configurations. If we take weighted arithmetic means of all the truncation term coefficient ratios from Figure 8 for truncation term orders up to 30 with weights derived from an exponential decay model with unit original amount and decay rate equal to −1 to account for decreasing contribution of higher order terms we obtain weighted average ratios of 1.91 and 3.99 respectively. These ratios are still within 20% of the respective ratios of Relative and Maximum Errors analytically verifying the results obtained by FEM modeling.

## 4. Discussion

The contribution of this paper is twofold. First, novel variable inter-ring distances CREs are proposed as opposed to all the previous research on CREs that, to the best of the authors’ knowledge, was based on the assumption of constant inter-ring distances. Laplacian estimates for variable inter-ring distances CREs are derived using a modified (4*n* + 1)-point method from Reference [28] for any given number of rings *n*. Second, accuracies of Laplacian estimates corresponding to constant, linearly increasing and linearly decreasing inter-ring distances TCRE and QCRE configurations are directly compared using FEM model analysis. FEM modeling results obtained in this paper are consistent with the previous FEM modeling results obtained for bipolar and tripolar CRE configurations only [10,11], as well as for multipolar CRE configurations up to the septapolar one [28] in terms of accuracy of Laplacian estimation increasing (Relative and Maximum Errors decrease) with an increase in the number of rings *n* and decreasing (Relative and Maximum Errors increase) with an increase in the diameter of the CRE. More importantly, obtained FEM modeling results suggest that increasing inter-ring distances CRE configurations may decrease Relative and Maximum Errors resulting in more accurate Laplacian estimates compared to respective constant inter-ring distances CRE configurations. For currently used TCREs the truncation error may be decreased more than two-fold while for QCREs more than six-fold decrease is expected. These results are verified analytically based on our hypothesis that the ratios of constant inter-ring distances truncation term coefficient functions over the increasing inter-ring distances truncation term coefficient functions (as well as of decreasing inter-ring distances truncation term coefficient functions over constant inter-ring distances truncation term coefficient functions) for TCRE and QCRE configuration will be comparable to the respective ratios of Relative and Maximum Errors obtained using the FEM model. The type of analysis that was used to confirm our hypothesis providing a new instrument for verification of FEM modeling results would not have been feasible in our previous works. For example, in Reference [28], where multipolar CRE configurations ranging from bipolar (*n* = 1) to septapolar (*n* = 6) were compared using FEM modelling, Laplacian estimates for different CRE configurations had different numbers of truncation terms (one truncation term less for each additional concentric ring causing an increase in Laplacian estimation accuracy) which made analytical comparison of truncation term coefficients for different CRE configurations infeasible. In this study proposed variable inter-ring distances CREs have the same numbers of rings and, therefore, the same numbers (and orders) of truncation terms in respective Laplacian estimates as their constant inter-ring distances counterparts which allowed us to quantify the expected improvement in estimation accuracy analytically. Therefore, this paper provides a comprehensive theoretical basis for variable inter-ring distances CREs, as well its validation via analytically verified FEM modeling.

Biomedical significance of CREs is related to the fact that errors presented in this manuscript translate directly into more accurate surface Laplacian estimates which is of critical importance since, for example, in applications to EEG it has been shown to alleviate the blurring effects enhancing the spatial resolution and selectivity, and reduce the reference problem [4,5,6]. This is why several methods were proposed for Laplacian estimation through interpolation of potentials on a surface and then estimating the Laplacian from an array of conventional (single pole) disc electrodes [35,36,37]. Since only CREs allow estimating Laplacian directly at each electrode instead of combining the data from an array of conventional disc (single pole) electrodes, further improving the accuracy of Laplacian estimation via variable inter-ring distances CREs may be critical to the advancement of noninvasive electrophysiological electrode design with application areas not limited to electroencephalography, electrocardiography, and electromyography. In particular, since “negative Laplacian is approximately proportional to cortical (or dura) surface potential” [38], every application currently utilizing Laplacian signals such as, for example, tEEG [10,11,12,13,14,15,16,17,18,20,21] may benefit from more accurate Laplacian estimation since it improves estimation of the cortical potentials. Moreover, other potential advantages of variable inter-ring distances CREs need to be investigated including, for example, improved control of the electric field used for seizure attenuation compared to the one that current transcranial focal stimulation applied via constant inter-ring distances TCREs can offer [17,18,19,20]. It should be noted that variable inter-ring distances CREs do not cause any inherent growth in the size of the electrode compared to their constant inter-ring distances counterparts since all CRE configurations considered and modeled had the same dimensions. Neither do they cause an inherent growth in computational complexity since null space of matrices from Equations (5), (7), and (8) can be found offline for any given *n* with the preamplifier board calculating the Laplacian estimate as the linear combination of differences of potentials from each of the *n* rings and the central disc respectively using this null space vector as the vector of coefficients. Finally, no inherent growth is caused in the number of amplifier channels since surface Laplacian estimate calculated by the preamplifier board is the only signal sent to the amplifier for each CRE. Therefore, variable inter-ring distances CREs are not expected to have any adverse effects on signal acquisition or implementation and complexity of the related hardware.

Further investigation is needed to confirm the obtained results. The plan for future work includes several directions and is based on limitations of the current study. The main limitation of both the proposed (4*n* + 1)-point method and the FEM model is that the width of the concentric rings and the diameter of the central disc are not taken into account and therefore cannot be optimized. To pursue our ultimate goal of being able to determine optimal CRE designs for specific applications these two parameters need to be included into future modifications of the (4*n* + 1)-point method and into the FEM model along with the currently included number of rings, size of the electrode, and, as proposed in this study, inter-ring distances. Another limitation is that while this study proposes variable inter-ring distances CREs for the first time, only linearly increasing and linearly decreasing inter-ring distances are considered. The solution to the general inter-ring distances optimization problem is likely to result in nonlinear relationship which is why solving this general problem is the second direction of the future work. Third direction is to create prototypes of variable inter-ring distances CREs with 2 and more rings and test them on real life data, both phantom and from human subjects. This direction is critical since obtained results suggest that variable inter-ring distances CREs may result in more accurate Laplacian estimates. This raises the question of how small can the distances between concentric rings get before partial shorting due to salt bridges becomes significant enough to affect Laplacian estimation. Moreover, these prototypes would allow investigating the translation of Relative and Maximum Errors assessed in this study into improvement of spatial selectivity, signal-to-noise ratio, mutual information, *etc.*, the same way it was investigated for tEEG compared to EEG with conventional disc electrodes [12]. Prototyping techniques envisioned include using both rigid substrates for nondisposable CREs (e.g., gold-plated copper on biocompatible dielectric) [10,11,12,13,14,15,16,17,18,19,20,21,22,24] and flexible substrates for disposable CREs (e.g., silver paste on polyester film) [23,25,26,27,39]. A comparative analysis of flexible CRE manufacturing techniques including screen-printing, inkjet, and gravure is available in Reference [39].

## 5. Conclusions

With tripolar concentric ring electrodes gaining increased recognition in a wide range of applications due to their unique capabilities this study assesses the potential of novel variable inter-ring distances concentric ring electrodes. Results of mathematical analysis and finite element method modeling for tripolar and quadripolar concentric ring electrode configurations are consistent in suggesting that increasing inter-ring distances concentric ring electrodes may offer more accurate Laplacian estimation compared to their constant inter-ring distances counterparts.

## Abbreviations

The following abbreviations are used in this manuscript:

EEG	Electroencephalography
CRE	Concentric Ring Electrode
TCRE	Tripolar Concentric Ring Electrode
QCRE	Quadripolar Concentric Ring Electrode
FPMtEEG	Five-Point MethodLaplacian Electroencephalography via Tripolar Concentric Ring Electrode
FEM	Finite Element Method
